# Effect of Audiovisual Cardiopulmonary Resuscitation Feedback Device on Improving Chest Compression Quality

**DOI:** 10.1038/s41598-019-57320-y

**Published:** 2020-01-15

**Authors:** Chia-Ying Lin, Shao-Hsuan Hsia, En-Pei Lee, Oi-Wa Chan, Jainn-Jim Lin, Han-Ping Wu

**Affiliations:** 1 Division of Pediatric Critical Care Medicine, Department of Pediatrics, Chang Gung Memorial Hospital at Linko, Kweishan, Taoyuan Taiwan; 2 College of Medicine, Chang Gung University, Taoyuan, Taiwan; 3 Department of Pediatric Emergency Medicine, Children’s Hospital, China Medical University, Taichung, Taiwan; 4 Department of Medical Research, Children’s Hospital, China Medical University, Taichung, Taiwan; 5 Department of Medicine, School of Medicine, China Medical University, Taichung, Taiwan

**Keywords:** Preventive medicine, Disease-free survival

## Abstract

The one month survival rate after out-of-hospital cardiac arrest (OHCA) in the paediatric population remains low. Improving survival in paediatric OHCA by enhancing the quality of cardiopulmonary resuscitation (CPR) is important. In this study, we aimed to analyse the factors associated with CPR quality by using a real-time feedback device. Participants were prospectively divided into 4 groups: paediatric research fellows, paediatric residents, medical students (clerks), and paediatric critical care nurses. Then, the participants were asked to perform 5 cycles of CPR on a paediatric simulation manikin without prompts from feedback devices, and to repeat another 5 cycles of CPR after education with the 2015 paediatric advanced life support guidelines. A total of 75 participants were evaluated. In the overall analysis, an improvement in the percentage of participants meeting the target compression rate was observed (from 49.82% to 71.23%, *P * < 0.001). The percentage of participants achieving the target compression depth improved from 73.77% to 85.63% (*P* = 0.005). Among the 4 groups, the residents showed the most significant improvement in both compression rate (from 48.41% to 86.57%, *P* < 0.001) and compression depth (from 63.50% to 95.57%, *P* < 0.001). Inappropriate rate was a more important factor resulting in inadequate CPR performance than inappropriate depth. An excessive compression rate was also a common problem. In conclusions**, t**he real-time CPR feedback device may help clinical physicians and nurses in improving the quality of chest compression. Excessive CPR compression rate may be a major cause of inadequate CPR performance.

## Introduction

Previous studies have shown that the 1-month survival rate among children who experienced out-of-hospital cardiac arrest (OHCA) remains very low (5.2–12.8%). The quality of cardiopulmonary resuscitation (CPR) performance may be the key factor affecting survival in patients who had OHCA^1^. According to the 2015 American Heart Association (AHA) Guidelines Update for CPR and Emergency Cardiovascular Care, a high-quality CPR involves appropriate chest compression rate, proper chest compression depth, complete chest recoil, minimisation of compression interruptions, and avoidance of excessive ventilation. Appropriate chest compression creates blood flow primarily by increasing intrathoracic pressure and directly compressing the heart, which results in blood flow and oxygen delivery to the heart, brain, and other vital organs. Complete chest recoil ensures adequate coronary perfusion pressure and myocardial blood flow^2^. Poor-quality CPR performance that does not meet the published guideline recommendations is not uncommon, even when CPR is performed by well-trained staff^3,4^.

In previous studies, poor CPR during in-hospital cardiac arrest and OHCA involved shallow and slow chest compressions, prolonged interruptions, and high ventilation rates^3,5^. By assessing CPR performance and providing real-time compression information, a CPR feedback device may be helpful in improving the quality of CPR^6,7^. During CPR training, learners who used devices that provided corrective feedback had improved compression rate, depth, and recoil compared with learners who performed CPR without the use of feedback devices^8^. According to the above benefits, the 2015 AHA guidelines suggest the use of a CPR feedback device when available (recommendation class IIb, evidence level C). The aim of this study was to evaluate the effectiveness of a feedback device on improving compression quality to meet the 2015 AHA paediatric basic life support (BLS) targets and to analyse the chest compression quality performed by paediatric care staff. In addition, to enhance the future training efficacy of paediatric advanced life support (PALS) and for the purpose of problem-based learning, we aimed to explore the common problems in paediatric chest compression.

## Materials and Methods

### Setting

This was a single-centre study of prospectively collected observational data, and a manikin study. The study was conducted in a tertiary care children’s hospital. The study was approved by the Institutional Review Board of the Chang Gung Memorial Hospital. All methods were performed in accordance with the relevant guidelines and regulations. The data were collected, reviewed, de-identified, and anonymously analyzed by the authors, and written informed consent was obtained from all participants.

### Study design and participants

The participants were divided into (i) paediatric research fellows, (ii) paediatric residents, (iii) fifth-year medical students (clerks), and (iv) paediatric critical care nurses at the paediatric intensive care unit. Residents were then divided into 3 subgroups according to seniority. Except for medical students who had been trained in only BLS, all others had been trained in PALS and had received certifications within the validated period. The study was conducted inside the paediatric intensive care unit. All participants were first asked to perform 5 cycles of CPR (1 cycle of 30 compressions with 2 breaths) on a paediatric simulation manikin (Little Junior™ child CPR training manikin; Laerdal Medical Canada, Toronto, Canada) without prompts from feedback devices. The participants were reminded that the simulated compression was intended to be performed on a child. After the compression, a 1-min break was allowed; then, a feedback device was introduced by a research fellow in paediatric critical care subspecialty who had trained and is certified in 2015 AHA PALS. The participants then performed another 5 cycles of CPR on the manikin according to the CPR targets of the 2015 AHA PALS, which included a compression rate of 100–120/min and a compression depth of around 5 cm.

The compression parameters displayed on the CPR dashboard of the feedback device included the (i) value of the compression rate, (ii) value of the compression depth, and (iii) extent of chest release (in the release bar, once complete release of the chest is properly done, the bar will fill all the way to the top). The participants were asked to look at the dashboard and adjust their compressions accordingly.

We attempted to quantify the extent of performance enhancement in terms of compression rate by using the following formula:

[targeted rate (%) with feedback – targeted rate (%) without feedback]/targeted rate (%) without feedback

The ‘targeted rate (%)’ represents the percentage of the target compression rate.

Similarly, the extent of performance enhancement in terms of compression depth was interpreted using the following formula:

[targeted depth (%) with feedback – targeted depth (%) without feedback]/targeted depth (%) without feedback

Medical students and doctors (residents and fellows) were divided into 2 subgroups with 1 group trained only in BLS (all of them were clerks) and another group trained/certified in PALS. The 2 subgroups were selected for quality analysis of original compression (compression without feedback prompts). The mean chest compression release velocity (CCRV) was stratified into 3 gradients: grade 1, ≥400 mm/s; grade 2, 300–399.9 mm/s; and grade 3, <300 mm/s.

### Data collection

Data were collected between April 2017 and March 2018. An electrocardiogram monitor/defibrillator with an accelerometer that provides real-time audio-visual feedback (R Series; ZOLL Medical, Chelmsford, MA, USA) was used. Adult-type electrodes were used for data collection and feedback information display (adult-type electrodes are suitable for children >8 years old or weighing >25 kg). The data of compression rate and depth were uploaded to Rescue Net Code Review software (ZOLL Data Systems; Broomfield, CO, USA) and analysed. Although chest release information could be displayed on the dashboard while performing chest compression, it was not available for data collection and further analysis. The mean CCRV, which could not be displayed during chest compression, was documented and analysed instead.

### Statistics

Descriptives of numerical data were presented as mean ± standard deviation, median (interquartile range), and n (%) for categorical data, when appropriate. The Mann-Whitney *U*-test was used for comparison of continuous variables between the groups. The comparison of compression depth and rate between CPR without and with feedback among the same population was done using a paired t-test. The extent of performance enhancements in both groups was analysed using 1-way analysis of variance, and Scheffe post-hoc test was performed. An independent-sample t-test was used for the comparison of compression rate and depth between subgroups trained with or without PALS. Differences between the groups were tested with the Pearson chi-square test for discrete variables such as CCRV. Statistical significance was set at *P* < 0.05. All statistical analyses were performed using the SPSS software (version 22.0; SPSS Inc., Chicago, IL, USA).

### Consent for publication

All authors have reviewed and approved the manuscript for publication.

### Ethics approval and consent to participate

The study protocol was approved by the Institution Review Board and ethics committee of Chang-Gung Memorial hospital.

## Results

**Figure 1:**
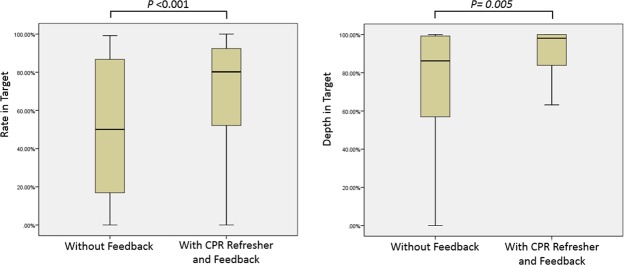
Compression rate and depth in targeted range with and without the aid of feedback device.

A total of 75 personnel participated in the study including 19 paediatric fellows, 27 paediatric residents, 14 clerks, and 15 paediatric critical care nurses. The age of the participants was between 20 and 39 years. The number of male and female participants was 24 (32%) and 51 (68%), respectively. Of them, 14 participants (18.7%) were trained in only BLS and 61 participants (81.3%) had PALS certification within the validity period. With the use of audio-visual feedback, the overall percentage of participants meeting the target mean compression rate improved from 49.82% to 71.23% (P < 0.001). The overall percentage of participants meeting the target mean compression depth improved from 73.77% to 85.63% (P = 0.005). Statistically significant differences were found both in the compression rate and compression depth with the aid of the feedback device (Fig. 1).

**Figure 2:**
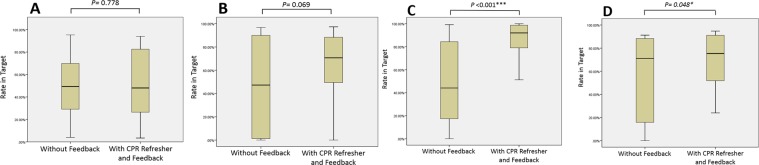
Compression rate in targeted range with and without the aid of feedback device by groups; (**A**) the nurse group, (**B**) the clerk group, (**C)** the resident group, (**D**) the fellow group.

**Figure 3:**
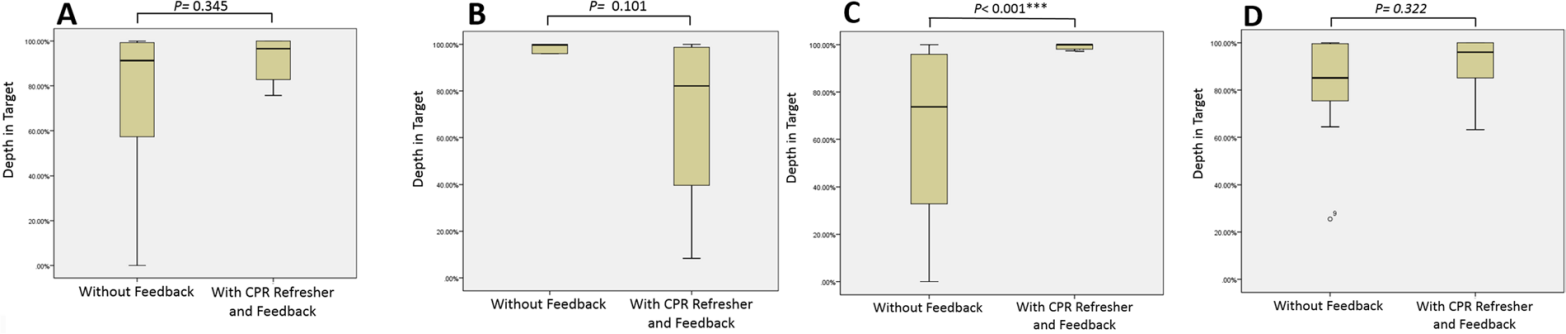
Compression depth in targeted range with and without the aid of feedback device by groups; (**A**) the nurse group, (**B**) the clerk group, (**C**) the resident group, (**D**) the fellow group.

The performance of CPR in the 4 groups was analysed individually with and without audio-visual feedback, and the results are shown in Table 1. For the compression rate, both the residents and fellows showed significant improvements in compression rate with feedback. However, the nurse and clerk groups showed little improvements with feedback, with no significant differences (Fig. 2). For the compression depth, there was a trend of improvement except for the clerk group. There was a significant improvement in the residents in meeting the target compression depth (Fig. 3). In addition, in the resident subgroup, the first-year, second-year, and third-year residents all showed significant improvements in achieving appropriate chest compression rate and depth with feedback (both *P* < 0.05) (Table 2).

**Table 1:** Percentages of Participants Meeting the Target Ranges of Compression Rate and Depth With and Without the Aid of Feedback Device by Groups.

	Without Feedback	With CPR Refresher and Feedback	*P*value
**Percentage meeting the target rate (%)**			
Nurses (n = 15)	49.3 (27.8–72.5)	48.0 (25.9–85.2)	0.778
Clerks (n = 14)	47.2 (1.28–89.9)	70.6 (44.1–89.2)	0.069
Residents (n = 27)	44.1 (16.8–86.8)	92.0 (77.6–99.1)	<0.001*
Fellows (n = 19)	71.1 (8.0–89.9)	75.3 (50.7–91.3)	0.048*
**Percentage meeting the target depth (%)**			
Nurses (n = 15)	91.3 (35.6–99.3)	96.6 (75.7–100.0)	0.345
Clerks (n = 14)	99.7 (91.8–100.0)	82.1 (38.4–98.7)	0.101
Residents (n = 27)	73.7 (29.9–97.0)	100.0 (98.1–100.0)	<0.001*
Fellows (n = 19)	85.1 (74.3–100.0)	96.0 (82.3–100.0)	0.322

Data are median (interquartile range). P < 0.05 significant.

CPR, cardiopulmonary resuscitation.

**Table 2:** Percentages of Participants Meeting the Target Ranges of Compression Rate and Depth With and Without the Aid of Feedback Device by Resident Groups.

	Without Feedback	With CPR Refresher and Feedback	*P* value
Percentage meeting the target rate (%)	Mean (SD)	Mean (SD)	
First-year residents (n = 9)	27.5 (33.4)	81.9 (16.3)	0.011*
Second-year residents (n = 12)	56.9 (33.4)	90.3 (12.8)	0.008**
Third-year residents (n = 6)	62.6 (37.0)	86.0 (19.6)	0.028*
**Percentage meeting the target depth (%)**			
First-year residents (n = 9)	63.9 (33.7)	89.9 (16.8)	0.025*
Second-year residents (n = 12)	63.4 (37.7)	98.1 (4.8)	0.003**
Third-year residents (n = 6)	62.9 (40.5)	98.9 (2.4)	0.028*

CPR, cardiopulmonary resuscitation; SD, standard deviation.

**Figure 4:**
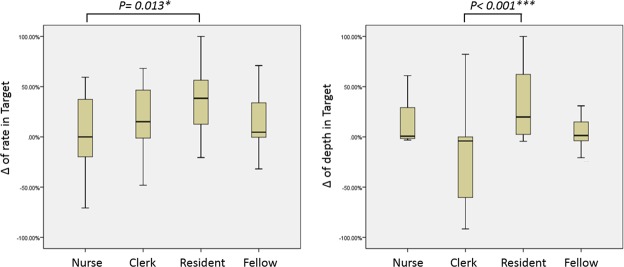
The Extent of Enhanced Performance of Compression Rate and Depth by groups.

**Figure 5:**
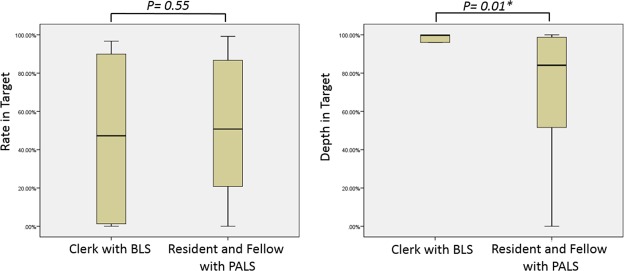
Rate and depth in targeted range of naïve chest compression by groups.

In the results shown in Table 3 and Fig. 4, the resident group had the highest percentage of participants meeting the target rate (%). The percentage of participants meeting the target mean compression rate in the BLS-trained group was 45.23% ± 40.42%, and that in the PALS-trained group was 50.88% ± 33.87%. The percentage of participants meeting the target mean compression depth in the BLS-trained group was 84.92% ± 33.08%, whereas that in the PALS-trained group was 71.20% ± 33.14% (*P* < 0.05) (Fig. 5). In addition, significantly increased mean CCRV was observed in the resident group with feedback aid (*P* < 0.05) (Table 4). Moreover, this phenomenon was especially obvious in the subgroup of senior residents (Table 5).

**Table 3:** Extent of Performance Enhancement in Compression Rate and Depth by Groups.

	Nurses (n = 15)	Clerks (n = 14)	Residents (n = 27)	Fellows (n = 19)	*P* value
Enhancement in meeting the target rate^a^, median (IQR)	0.0 (−29.5–44.0)	15.1 (−2.0–46.9)	38.4 (9.3–57.4)	5.2 (−0.8–42.7)	0.013^c^ *
Enhancement in meeting the target depth^b^, median (IQR)	0.7 (−1. = 9–43.5)	−4.1 (−61.6–0.2)	19.8 (2.0–70.1)	0.8 (−4.0–14.9)	<0.001^d^ *

^a^Percentage of ‘compression rate in the targeted range with skill refresher and audiovisual feedback’ minus ‘original compression rate in the targeted range’.

^b^Percentage of ‘compression depth in the targeted range with skill refresher and audiovisual feedback’ minus ‘original compression depth in the targeted range’.

^c^Statistically significant difference between the nurse and resident groups, *P* = 0.017.

^d^Statistically significant difference between the clerk and resident groups, *P* = 0.004.

CPR, cardiopulmonary resuscitation; IQR, interquartile range.

**Table 4:** Release Velocity With and Without the Aid of a Feedback Device by Groups.

Release Velocity (mm/s)	Without Feedback n (%)	With CPR Refresher and Feedback n (%)	*P* value
Nurses, total n = 15			0.091
Grade 1 (≥400)	4 (26.7%)	0 (0.0%)	
Grade 2 (300–399.9)	8 (53.3%)	12 (80.0%)	
Grade 3 (<300)	3 (20.0%)	3 (20.0%)	
Clerks, total n = 14			0.231
Grade 1 (≥400)	1 (7.1%)	4 (28.6%)	
Grade 2 (300–399.9)	12 (85.7%)	8 (57.1%)	
Grade 3 (<300)	1 (7.1%)	2 (14.3%)	
Residents, total n = 27			<0.001*
Grade 1 (≥400)	12 (44.4%)	0 (0.0%)	
Grade 2 (300–399.9)	12 (44.4%)	17 (63.0%)	
Grade 3 (<300)	3 (17.1%)	10 (37.0%)	
Fellows, total n = 19			0.744
Grade 1 (≥400)	6 (31.6%)	5 (26.3%)	
Grade 2 (300–399.9)	11 (57.9%)	13 (68.4%)	
Grade 3 (<300)	2 (10.5%)	1 (5.3%)	

CPR, cardiopulmonary resuscitation.

**Table 5:** Release Velocity With and Without the Aid of a Feedback Device by Resident Seniority.

Release Velocity (mm/s)	Without Feedback n (%)	With CPR Refresher and Feedback n (%)	*P*-Value
First-year residents, total n = 9			0.056
Grade 1 (≥400)	3 (33.3)	0 (0.0)	
Grade 2 (300–399.9)	5 (55.6)	4 (44.4)	
Grade 3 (<300)	1 (11.1)	5 (55.6)	
Second-year residents, total n = 12			0.042*
Grade 1 (≥400)	5 (41.7)	0 (0.0)	
Grade 2 (300–399.9)	5 (41.7)	8 (66.7)	
Grade 3 (<300)	2 (16.7)	4 (33.3)	
Third-year residents, total n = 6			0.043*
Grade 1 (≥400)	4 (66.7)	0 (0.0)	
Grade 2 (300–399.9)	2 (33.3)	5 (83.3)	
Grade 3 (<300)	0 (0.0)	1 (16.7)	

CPR, cardiopulmonary resuscitation.

## Discussion

In this study, we found that a real-time CPR feedback device could help clinic staff in improving the quality of CPR. Significant benefits of using the feedback device were observed in the resident and fellow groups, especially in the resident group. Compared with the nurse and clerk groups, the resident and fellow groups were relatively more experienced and had a certain familiarity with the hands-on practice of CPR techniques. The prompts from the feedback device contributed an add-on effect to performance enhancement. The residents are the key members involved in paediatric primary care, which probably explains why they were the most enthusiastic group in terms of study participation and about receiving feedback information. In the analysis of original compression, improved skill performance was noticed along with the seniority of residentship.

Previous studies had found that the compression rates were generally too slow^9^. However, our study showed an opposite result, in that excessive compression rates were commonly observed. Excessive compression rates may result in insufficient compression depths and eventually lead to poor performance quality^10^. From our results, although all 4 groups showed improvements in chest compression, the extents of the progress in certain groups did not meet our expectations. In the post-test interview of our participants, some stated that if chest compression rates and depths remained stable within the target ranges, it would be easy to maintain the rate and depth within the ideal ranges. In contrast, if the rate and depth fell out of the target ranges, it would be difficult to maintain them within the ideal ranges. Once rate misjudging occurs, chest compression deceleration continues and may eventually lead to excessive deceleration. The same situation may also occur when dealing with compression depth. In addition, distraction may be one of the reasons for inadequate performance. Some participants claimed that it is challenging to pay attention to all 3 of the chest compression items at the same time. Moreover, the participants were nervous about the CPR simulation and were unfamiliar with the feedback device, and these issues may have had an impact, especially for the junior staff. Therefore, performance coaching might have a role in performance enhancement. The role of a ‘CPR coach’ is different from that of a CPR leader. The CPR coach supervises performance of chest compression and provides immediate feedback to ensure that a high-quality CPR is performed. Previous studies found that institutions with CPR coaches in addition to feedback devices have greater adherence to the AHA guidelines for chest compression rate and depth. A higher rate of return of spontaneous circulation was documented in those hospitals that routinely use CPR coaches^11,12^.

In the AHA guidelines, the suggested compression depth is no longer as deep as possible. Although a compression depth of at least 2 in (5 cm) in adult CPR was recommended in the 2010 AHA guidelines, the 2015 updated recommendation incorporates evidence about the potential for an upper threshold of compression depth (2.4 in or 6 cm), beyond which complications may occur. At present, there is no precise recommendation on the upper limit of the compression depth for the paediatric population. However, it is reasonable to speculate that an appropriate interval of paediatric compression depth exists, which should be less than the adult interval of 1 cm. Identification of compression depth and its upper limit may be challenging without the aid of feedback prompts, thus highlighting the importance of a feedback device.

Recently, a new performance metric of CCRV has been discussed. Most CPR feedback devices use accelerometers, which cannot directly measure leaning forces. Monitoring of leaning forces would require the use of a force sensor. The force sensor is relatively larger and more expensive than an accelerometer. The challenges in the direct measurement of leaning or recoil result in the use of indirect measurements, such as CCRV calculated from the accelerometry signal, to assess the performance of chest recoil^13–16^. An improved survival and favourable neurologic outcomes from hospital discharge after OHCA had been documented^14,15^. Survival after adult OHCA varied significantly with CCRV, as did favourable neurologic outcomes. Fast CCRV was associated with a higher survival rate after OHCA in adults, compared with slow and moderate CCRV. We speculated that slow CCRV was also associated with slow chest compression rates. Owing to the lack of paediatric data, we stratified CCRV into the same gradients and defined them as grade 1 (≥400 mm/s), grade 2 (300–399.9 mm/s), and grade 3 (<300 mm/s) to represent fast, moderate, and slow CCRV, respectively. We observed significantly increased mean CCRV in the resident group with feedback aid. Moreover, this phenomenon was especially obvious in the senior resident subgroup.

### Study limitations

Our study did have some limitations. First, we analyzed a relatively small group of tested persons at a single medical canter. There may be a risk of missing data or information bias. Second, the study is only conducted in one paediatric simulation manikin (Little Junior™ child CPR training manikin) which is classified as a child and the recommended compression depth is 5 cm. It should be noted that the chest-compression depth are different based on different pediatric age. According to 2015 AHA guidelines, a compression depth of one third the anterior-posterior diameter of the chest is recommended. The recommended compression depth is 1.5 inches (4 cm) for infant and 2 inches (5 cm) for children (Class IIa). And the recommended compression depth for adolescent is 5–6 cm (Class I)^17^. Therefore further studies for analysing the chest compression quality performed on different simulation manikins (infant and adolescent) may be warranted.

## Conclusions

The real-time CPR feedback device may be helpful in improving chest compression compliance. In addition, residents and fellows could benefit from the device more than clerks and nurses. Inadequate CPR performance more commonly results from inappropriate CPR rates than inappropriate CPR depths, and excessive CPR rates are common.
